# Molecular imaging-challenges, opportunities and caveats

**DOI:** 10.4103/0971-6203.79684

**Published:** 2011

**Authors:** A. K. Shukla

**Affiliations:** Department of Nuclear Medicine, Sanjay Gandhi Postgraduate Institute of Medical Sceinces, Lucknow – 226 014, UP, India. E-mail: akshukla@sgpgi.ac.in

The classical form of diagnostic imaging includes X-ray-based imaging systems, magnetic resonance imaging (MRI), and sonographic imaging. Such imaging systems in a given clinical situation document the findings which reflect the end effect of molecular/biochemical changes associated with a particular disease condition. The lack of information at molecular or cellular level as a precursor to a disease condition resulted into emergence of “Molecular Imaging.” This approach is expected to provide an imaging tool that enables visualization, characterization, and measurement of biological events at molecular and cellular level both in human being and other living species. Broadly speaking, molecular imaging investigates molecular signature of the disease through measurement and characterization of biological processes in a molecular or cellular compartment and thus enables opportunities to replace population-based treatment methods by personalized genotype/phenotype-adapted concepts.[1] If this opportunity is further extrapolated to current practices in radiation oncology, it would offer more reliable target volume definition and more accurate specification of tumor heterogeneity apart from effective assessment of tumor response and normal tissue reactions.

In other words, cell growth or destruction responsible for biochemical changes can be visualized and quantified by using molecular imaging techniques so as to delineate disease pattern specific to an individual(s) in its early stages. Such findings would score in terms of personalized medicine based on physiological and/or biochemical changes particularly when it is well known that anatomical/structural changes in a given disease condition appear much later than the biochemical changes.

A wide range of fast emerging techniques rely on visualization of biological events or biochemical changes in a biological system. These include molecular nuclear medicine, magnetic resonance spectroscopy (to identify proteins produced during the progression of the disease), and bioluminescence imaging. These techniques can also be successfully deployed to differentiate between rapidly proliferating tumor cells and normal cells. In addition, these techniques can be used to depict angiogenesis of formation of new blood vessels. Thus the quantification of cellular changes with time enables characterization of disease process.

Nuclear medicine with latest developments in terms of positron emission tomography (PET) radiopharmaceuticals[2] and fusion imaging with computerized tomography (CT) or magnetic resonance imaging (MRI) offers greater opportunities and plays a vital role in clinical practice of molecular imaging. The current trends are suggestive of the fact that mere interpretation of nuclear images is no longer an objective of a clinician and it is equally important to extract relevant data from the acquired images so as to predict biochemical and physiological changes at molecular or cellular level. For instance, measurement of a standard uptake value (SUV) on a PET image requires knowledge of timing of an imaging procedure, patient’s physical parameters like height and weight as well as accumulated counts in the given region of interest (ROI). The relative activity concentration can be measured/calculated in a particular target area and simultaneously time activity curve (TAC) can also be generated together with an SUV. The TAC so generated would be of immense clinical utility to effectively monitor the disease progression with time. However, utmost care and precision need to be exercised to ensure reproducibility and accuracy of such image-based quantitative measurements. This feature of nuclear medicine imaging has assigned it a characteristic and dominant role in the entire gamut of molecular imaging techniques. Besides nuclear medicine, the other tools which appear promising include optical imaging, MR spectroscopy, micro bubbles sonography, and functional MRI. The frontline concepts relating to hybrid imaging modalities (including single photon emission tomography (SPECT), CT, PET/CT and PET/MRI) have futuristic goals and incorporate promising characteristics of co-registration of functional and anatomical images.[3]

Thus, the hybrid imaging owing to its capability to provide physiostructural information shall continue to be the cornerstone in molecular imaging.[4] It would also be important to mention here that the hybrid imaging with PET and MRI if successfully launched would be a milestone in the field of molecular imaging and would promise advanced clinical applications in oncology, neurology, and cardiology. Apart from such unprecedented opportunities, whole body molecular imaging may also add new dimensions in clinical research as well as effectively contribute to patient care through a unique approach of personalized medicine. The focus of nuclear medicine would therefore get transformed to obtaining target information at cellular/molecular level instead of interpretation of a standalone image with reference to pre-established patterns associated with a particular disease type. The use of an appropriate molecular probe or marker for the diagnosis of a particular disease in a particular patient would be the order of the day. Based on the information derived in this manner, various treatment options could be explored and exercised to deliver personalized medicine (right drug for the right disease in a right patient). It appears to be now a reality to identify non-responders and drastically reduce the overall cost of health care. It is with this view that the most accepted tool of molecular imaging (especially hybrid imaging) would get integrated with therapeutic protocols of personalized medicine. The tools of computational biology and bioinformatics would also be of immense utility in drug development and therapeutic protocols of personalized medicine.

In the era of personalized medicine molecular imaging with appropriate biomarkers/probe would initiate a concept of individualized imaging and hence individualized diagnosis as well as treatment.[5] Such treatment protocols would be evolved based on patient-specific molecular signature and specific biological processes involved in a disease process. The future of imaging therefore appears to have already skewed toward molecular imaging and hybrid imaging as such is expected to register phenomenal growth in near future to get integrated into the therapeutic protocols of evidence-based personalized medicine.
